# 731. Puerperal Sepsis Among Women with In-facility Births in Western Tanzania

**DOI:** 10.1093/ofid/ofab466.928

**Published:** 2021-12-04

**Authors:** Rachel Smith, Alicia Ruiz, Matthew Westercamp, Godson Maro, Florina Serbanescu

**Affiliations:** 1 Centers for Disease Control and Prevention, Decatur, GA; 2 CDC, Atlanta, Georgia; 3 Bloomberg Philanthropies, Dar es Salaam, Dar es Salaam, Tanzania

## Abstract

**Background:**

Puerperal sepsis is an important cause of maternal mortality worldwide. As access to emergency obstetric services expands in resource-limited settings, rapid recognition and treatment of sepsis, and prevention of nosocomial infections that might lead to sepsis, is critical. We describe puerperal sepsis cases among women with in-facility births in the Kigoma region of Tanzania.

**Methods:**

Demographic, obstetric history, pregnancy complication and outcome, as well as mortality data were collected for women who delivered in hospitals, health centers and dispensaries in the Kigoma region, Tanzania 2016 – 2018. Up to 3 maternal complications were recorded as free text. Puerperal sepsis included women where ‘sepsis’ was recorded as a complication during hospitalization. We calculated rates of puerperal sepsis and completed a descriptive analysis of patients.

**Results:**

203,604 women delivered infants in 197 participating facilities during the data collection period. Of these, 2228 (1.1%) had sepsis recorded, for an overall rate of 10.9 sepsis cases per 1000 deliveries. Although 48% of births occurred in dispensaries, sepsis complications were reported almost exclusively in hospitals and health centers (37.7 and 10.3 per 1000 deliveries, respectively). Sepsis rates varied across individual facilities, from 15.5 to 45.2 cases per 1000 deliveries in hospitals and 0 to 38.6 cases per 1000 deliveries in health centers. Women who developed sepsis had a median age of 25 (IQR 22 – 30) years and 1113 (56%) were nulliparous. 1763 (90%) of women who had sepsis delivered by caesarian delivery. Obstructed labor (827; 42%) was a common co-complication of sepsis; obstetric hemorrhage and uterine rupture were seen in 93 (5%) and 77 (4%) women with sepsis, respectively. 49 women with sepsis (3%) died prior to hospital discharge. Stillbirths and pre-discharge neonatal deaths complicated 107 (5%) and 74 (4%) deliveries to women with sepsis.

**Conclusion:**

In the Kigoma region of Tanzania puerperal sepsis frequently occurs in women with obstructed labor and caesarian delivery. Further evaluation of both facility-level and individual factors that contribute to the incidence of sepsis in this population, particularly those related to invasive procedures, is critical for early recognition and prevention. issue

**Disclosures:**

**All Authors**: No reported disclosures

